# An inflammation-related nomogram for predicting the survival of patients with non-small cell lung cancer after pulmonary lobectomy

**DOI:** 10.1186/s12885-018-4513-4

**Published:** 2018-06-26

**Authors:** Ying Wang, Xiao Qu, Ngar-Woon Kam, Kai Wang, Hongchang Shen, Qi Liu, Jiajun Du

**Affiliations:** 1 Institute of Oncology, Shandong Provincial Hospital Affiliated to Shandong University, 324 Jingwu Road, Jinan, 250021 People’s Republic of China; 2 Department of Thoracic Surgery, Shandong Provincial Hospital Affiliated to Shandong University, 324 Jingwu Road, Jinan, 250021 People’s Republic of China; 3 Department of Oncology, Shandong Provincial Hospital Affiliated to Shandong University, 324 Jingwu Road, Jinan, 250021 People’s Republic of China; 4 Department of Clinical Oncology, The University of Hong Kong, Laboratory block, 21 Sassoon, Pokfulam, Hong Kong, People’s Republic of China

**Keywords:** Non-small cell lung cancer, Inflammatory response biomarker, Nomogram

## Abstract

**Background:**

Emerging inflammatory response biomarkers are developed to predict the survival of patients with cancer, the aim of our study is to establish an inflammation-related nomogram based on the classical predictive biomarkers to predict the survivals of patients with non-small cell lung cancer (NSCLC).

**Methods:**

Nine hundred and fifty-two NSCLC patients with lung cancer surgery performed were enrolled into this study. The cutoffs of inflammatory response biomarkers were determined by Receiver operating curve (ROC). Univariate and multivariate analysis were conducted to select independent prognostic factors to develop the nomogram.

**Results:**

The median follow-up time was 40.0 months (range, 1 to 92 months). The neutrophil to lymphocyte ratio (cut-off: 3.10, HR:1.648, *P* = 0.045) was selected to establish the nomogram which could predict the 5-year OS probability. The C-index of nomogram was 0.72 and the 5-year OS calibration curve displayed an optimal agreement between the actual observed outcomes and the predictive results.

**Conclusions:**

Neutrophil to lymphocyte ratio was shown to be a valuable biomarker for predicting survival of patients with NSCLC. The addition of neutrophil to lymphocyte ratio could improve the accuracy and predictability of the nomogram in order to provide reference for clinicians to assess patient outcomes.

## Background

Lung cancer remains the leading cause of cancer-related death worldwide and 85% of lung cancers diagnosis are non-small cell lung cancer (NSCLC). Numerous studies investigated the prognostic factors in the early stage patients in order to establish a more efficient model to assess patient prognosis. In the seventh edition of the American Joint Committee on Cancer TNM classification, tumor extent, lymph node involvement and distant metastasis contributed significantly to individualized survival predictions [1]. In recent years, more studies reported that tumor characteristics were not the only determinants to predict the outcomes of patients with cancer. As inflammation emerged as a hallmark of cancer, inflammatory response biomarkers have shown to be promising prognostic factors for improving the predictive accuracy in cancer research. In 1986, Shoenfeld et al. demonstrated that high level of white blood cells in peripheral blood was associated with poor outcomes in patients who suffered from non-hematological malignancies [2]. Neutrophil to lymphocyte ratio [3–9], calculated by the ratio of absolute neutrophil counts to absolute lymphocyte counts in whole blood, was established by Walsh et al. who reported its potential prognostic value in colorectal cancer [10]. Additionally, derived neutrophil to lymphocyte ratio [5, 11, 12], lymphocyte to monocyte ratio [13, 14], platelet to lymphocyte ratio [3, 7] and systematic immune-inflammation index [15] were considered as potential systematic inflammatory response biomarkers for survival prediction. Although some articles have studied the prognostic or predictive value of these inflammatory response biomarkers, inflammation-related nomogram on NSCLC remains undefined.

Nomogram is a relative novel and convenient model to predict survivals of patients with cancer. It could generate an intuitive graph by integrating diverse determinant variables and reflect an individual probability of a clinical event. Postoperative nomograms can assist patients and physicians to get more information about the prognosis.

In this study, we have evaluated the prognostic values of various inflammatory response biomarkers and selected the most significant factors to establish our nomogram model. The established nomogram was compared with traditional TMN staging system to validate its effectiveness.

## Methods

From January 2006 to December 2011, 1454 patients with lung cancer (including adenocarcinoma or squamous cell carcinoma) who underwent surgery in Shandong Provincial Hospital Affiliated to Shandong University were retrospectively reviewed and consecutively selected. The clinical stages of all patients were identified according to the seventh edition TNM classification. The exclusion criteria included: Patients with incomplete clinical and pathological data; patients with distant metastasis or stage IV; Patients whose primary cancers were not lung cancer; Patients who received radiotherapy or chemotherapy before surgery. We reviewed the hospital records of 952 patients who met the criteria. All patients underwent lung resection and systematic lymph node sampling. Demographic data (age, gender), clinical characteristics (biochemical index, smoking history), histopathological results (pathological type, differentiation, pathological stage of tumor and involved lymph nodes according to TNM system staging), postoperative outcomes and survival data were collected and recorded. Tumor size was assessed using the longest diameter of the tumor. The information of tumor size, nodal metastases and distant metastasis were collected from the pathological and medical image reports.

### Ethics statement

All patients provided written informed consent for their information to be stored in the hospital database and used for research. Ethical approval was obtained from Provincial Hospital Affiliated to Shandong University ethics committee, and the study was carried out in accordance with the approved guidelines.

### Postoperative Treatment and Follow-up

All patients involved in our study were followed up from surgery to July 2014. The minimal follow-up period was 36.0 months (range, 1 to 92 months) and median follow-up time was 40.0 months. Routine examinations such as CT scan postoperatively were performed every 3 months for the first year, every 6 months for the second year and then once a year thereafter.

### Candidate biomarkers

The hematological variables were obtained from blood tests routinely performed 1–3 days before surgery. Inflammatory response biomarkers included: neutrophil to lymphocyte ratio, absolute neutrophil counts to absolute lymphocyte counts, lymphocyte to monocyte ratio, platelet to lymphocyte ratio and systematic immune-inflammation index, which were calculated in the analysis. Neutrophil to lymphocyte ratio is defined as the ratio of absolute neutrophil count to absolute lymphocyte count in whole blood. Absolute neutrophil counts to absolute lymphocyte counts is defined as the ratio of absolute neutrophil count to the absolute white cell count minus the absolute count of neutrophils in whole blood. Platelet to lymphocyte ratio is defined as the ratio of absolute platelet count to absolute lymphocyte count in whole blood. Lymphocyte to monocyte ratio is defined as the ratio of absolute lymphocyte count to the absolute monocyte count in whole blood. Systematic immune-inflammation index is defined as the results of the peripheral platelet count multiplied by neutrophil count and divided by lymphocyte counts in whole blood.

### Statistical analysis

Demographic characteristics were showed through descriptive statistics. Normally distributed continuous data was presented as mean ± standard deviation, while discrete data was presented as count and proportion. Overall survival (OS) was defined as the period from surgery to death or the last date of follow-up for patients alive. The optimal cut-off levels of neutrophil to lymphocyte ratio, absolute neutrophil counts to absolute lymphocyte counts, lymphocyte to monocyte ratio and platelet to lymphocyte ratio were obtained by ROC analysis based on OS. Survival curves were derived by the Kaplan-Meier method and were assessed by log-rank test univariately. A Cox proportional hazards model was used to conduct multivariate analysis, with a significance level set at two-sided 0.05. Multivariable stepwise Cox models were performed to select final variables for prognostic factors. Above steps were performed with the statistical software SPSS version 20.0.

Based on the results of the multivariable analysis, a nomogram was established by R 3.2.0 software (Institute for Statistics and Mathematics, Vienna, Austria) with the rms and survival package. Internal validation of the nomogram was conducted and it was subjected to 1000 bootstrap resamples. Then we compared this nomogram with traditional TNM system staging by Harrell’s concordance index (c-index) to validate the accuracy of the nomogram. After bias correction, calibration curves on 5-year OS were generated by comparison between the predicted survival and observed survival [16].

## Results

### Clinicopathological features

Totally 952 eligible NSCLC patients, 674 men and 278 women, were enrolled into this study, with a mean age of 59 years (range, 20 to 79 years old). The primary tumor size ranged from 3 to 130.0 mm with a mean size of 38.6 mm, while the pathologic T stage showed 300 patients were in pathologic T1, 515 in pathologic T2,79 in pathologic T3 and 58 in pathologic T4. According to TNM system staging, pathological N stages were divided into three levels, and among them there were 530 pathologic N0 patients, 204 pathologic N1 patients, 213 pathologic N2 patients and 5 pathologic N3 patients. There were 416 patients with squamous cell carcinoma and 536 patients with adenocarcinoma respectively. Regarding degree of tumor differentiation, 131 patients were identified as well differentiated, 676 patients were identified as moderately differentiated and 145 patients were identified as poorly differentiated. Among the enrolled patients, 772 patients had the smoking experience and 180 patients did not have the experience. There were 483 patients received adjuvant chemotherapy after surgery and 483 patients did not receive chemotherapy. The characteristic information based on neutrophil to lymphocyte ratio was shown in Table 1. The optimal cut-offs obtained from ROC curves of neutrophil to lymphocyte ratio, absolute neutrophil counts to absolute lymphocyte counts, lymphocyte to monocyte ratio and platelet to lymphocyte ratio and systematic immune-inflammation index were shown in Table 2. Patients were divided into groups on the basis of optimal cut-offs.

**Table 1:** The clinicopathological characteristics based on neutrophil to lymphocyte ratio

	Total(*n* = 952)	NLR	
		<3.1(*n* = 732)	>3.1(*n* = 220)
Gender			
Male	674	486	188
Female	278	246	32
Age	59(20–79)	59(20–79)	60(27–78)
Smoking history			
N	180	126	54
Y	772	606	166
pT category			
pT1	300	233	67
pT2	515	391	124
pT3	79	61	18
pT4	58	47	11
pN category			
pN0	530	416	114
pN1	204	150	54
pN2	213	163	50
pN3	5	3	2
Histology			
ADC	536	453	83
SCC	416	279	137
PGTD			
I	131	111	20
II	676	508	168
III	145	113	32
Chemotherapy			
N	469	371	98
Y	483	361	122

*pT category* pathologcial T category

*pN category* pathologcial N category

*ADC* adenocarcinoma

*SCC* squamous cell carcinoma

*PGTD* pathological grading of tumor differentiation

*NLR* neutrophil to lymphocyte ratio

**Table 2:** The optimal cut-off point based on OS

	Median values	Range	AUC	Cut-off
NLR	2.49	0.33–12.40	0.584	3.1
dNLR	0.68	0.21–9.79	0.423	0.499
PLR	140.43	31.22–450.00	0.553	170.58
LMR	4.72	0.66–195.00	0.428	3.53
SII	614.99	76.26–3954.03	0.582	781.82

*NLR* neutrophil to lymphocyte ratio

*dNLR* derived neutrophil to lymphocyte ratio

*PLR* platelet to lymphocyte ratio

*LMR* lymphocyte to monocyte ratio

*SII* systematic immune-inflammation index

### Independent prognostic factors screened for nomogram

**Fig. 1:**
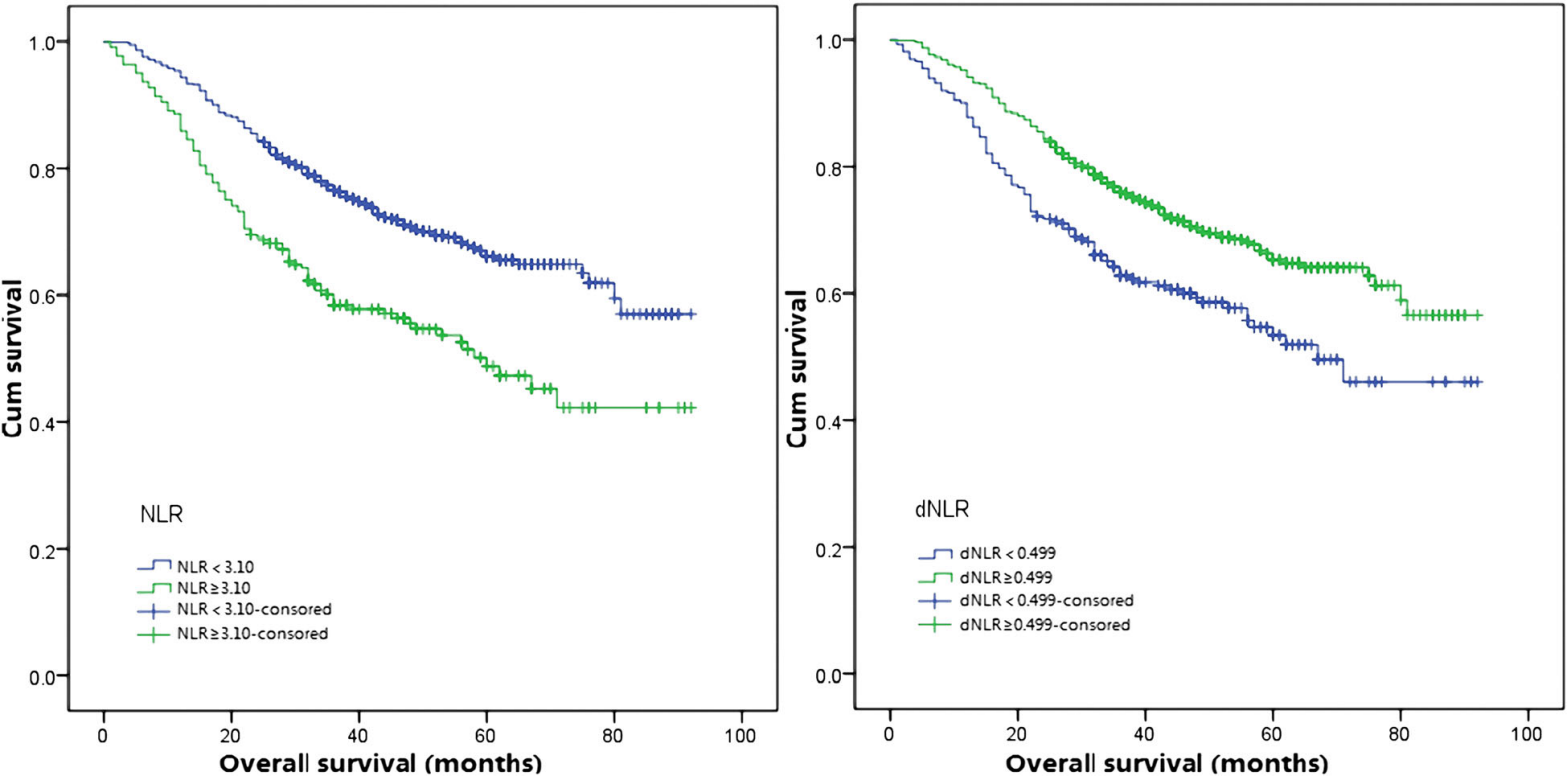
Kaplan-Meier curves for overall survival according to NLR and dNLR. NLR: Neutrophil to lymphocyte ratio; dNLR: Derived neutrophil to lymphocyte ratio

**Fig. 2:**
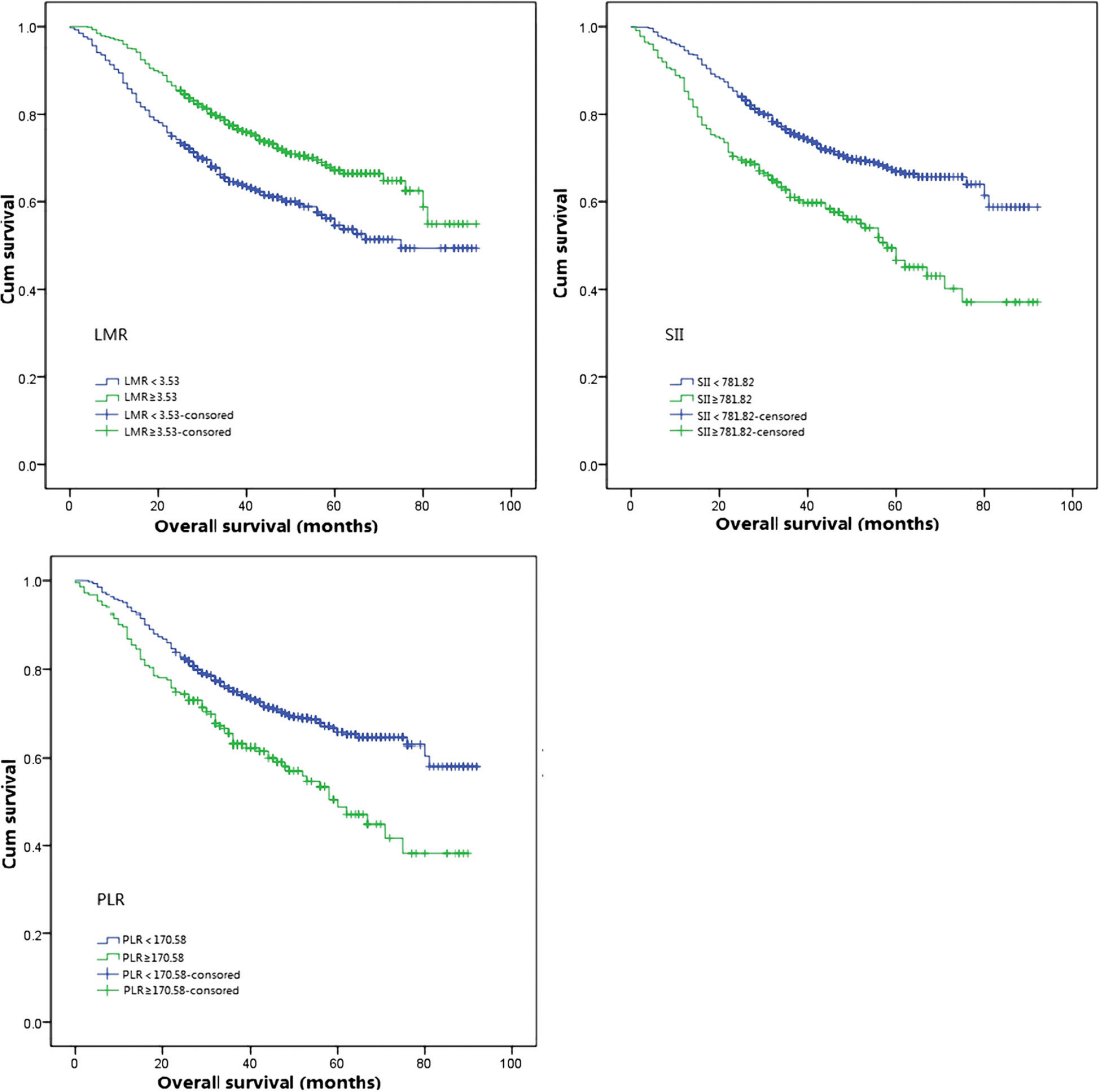
Kaplan-Meier curves for overall survival according to SII, LMR and PLR. PLR: platelet to lymphocyte ratio; LMR: lymphocyte to monocyte ratio; SII: systematic immune-inflammation index

Kaplan-Meier survival analysis was conducted to evaluate the relationship between inflammatory response biomarkers and survival outcomes. Patients were divided into two groups based on the optimal cutoffs of inflammatory response biomarkers (in Table 3),and all groups had significantly different survival ends(in Figs. 1 and 2). The univariate analysis indicated that age, neutrophil to lymphocyte ratio, absolute neutrophil counts to absolute lymphocyte counts, lymphocyte to monocyte ratio and platelet to lymphocyte ratio and systematic immune-inflammation index, pathologic T staging, pathologic N staging, tumor differentiation and smoking history were associated with OS (in Table 4). Multivariate analysis suggested that age, pathologic T and N staging, tumor differentiation, neutrophil to lymphocyte ratio were significantly associated with patients with reduced OS.

**Table 3:** Univariable analysis and cox proportional hazards regression analysis

Variable	Univariable analysis			Multivariable analysis		
	Hazard ratio	95% CI	*P*	Hazard ratio	95% CI	*P*
Age	1.388	1.112–1.733	0.004	1.649	1.306–2.081	<0.001
Gender						
Female	R					
Male	1.243	0.965–1.601	0.092			
Smoking history						
N	R					
Y	1.426	1.120–1.815	0.004	1.157	0.878–1.524	0.300
pT category						
T1–2	R			R		0.007
T3–4	2.030	1.558–2.645	<0.001	1.455	1.097–1.930	0.009
pN category						
N0	R		<0.001	R		<0.001
N1	2.414	1.808–3.22	<0.001	2.277	1.690–3.067	<0.001
N2	4.097	3.153–5.323	<0.001	4.233	3.216–5.570	<0.001
N3	6.170	1.953–19.493	0.002	5.121	1.530–17.144	0.008
Histology						
Histology ADC	R			R		
Histology CC	1.311	1.050–1.636	0.017	0.965	0.744–1.252	0.789
PGTD						
PGTD I	R		<0.001	R		0.008
PGTD II	0.174	0.091–0.332	<0.001	2.671	1.433–4.979	0.002
PGTD III	0.789	0.594–1.049	0.103	2.563	1.315–4.999	0.006
Chemotherapy						
N	R					
Y	1.166	0.934–1.457	0.175			
NLR						
<3.1	R					
>3.1	1.845	1.457–2.337	<0.001	1.648	1.010–2.687	0.045
dNLR						
<0.499	R					
>0.499	0.627	0.497–0.790	<0.001	1.470	0.936–2.309	0.095
PLR						
<170.58	R					
>170.58	1.636	1.284–2.085	<0.001	1.201	0.895–1.621	0.221
LMR						
<3.53	R					
>3.53	0.619	0.496–0.772	<0.001	1.020	0.770–1.351	0.890
SII						
<781.82	R					
>781.82	1.852	1.463–2.344	<0.001	1.412	0.955–2.090	0.084

*pT category* pathologcial T category

*pN category* pathologcial N category

*R* reference

*ADC* adenocarcinoma

*SCC* squamous cell carcinoma

*PGTD* pathological grading of tumor differentiation

*NLR* neutrophil to lymphocyte ratio

*dNLR* derived neutrophil to lymphocyte ratio

*PLR* platelet to lymphocyte ratio

*LMR* lymphocyte to monocyte ratio

*SII* systematic immune-inflammation index

**Table 4:** The survival data of subgroups according to inflammation response biomarkers

Groups	Cutoff	Patients	3-year OS	5-year OS	*P*
NLR					<0.001
<3.10		732	76.50%	66.10%	
>3.10		220	58.40%	48.80%	
dNLR					<0.001
<0.499		262	79.50%	62.80%	
>0.499		690	65.30%	53.40%	
PLR					<0.001
<170.58		738	75.00%	63.00%	
>170.58		214	65.70%	48.80%	
LMR					<0.001
<3.53		388	64.60%	77.60%	
>3.53		564	54.60%	57.20%	
SII					<0.001
<781.82		729	75.70%	61.00%	
>781.82		233	66.90%	46.70%	

*NLR* neutrophil to lymphocyte ratio

*dNLR* derived neutrophil to lymphocyte ratio

*PLR* platelet to lymphocyte ratio

*LMR* lymphocyte to monocyte ratio

*SII* systematic immune-inflammation index

### Prognostic nomogram on OS

**Fig. 3:**
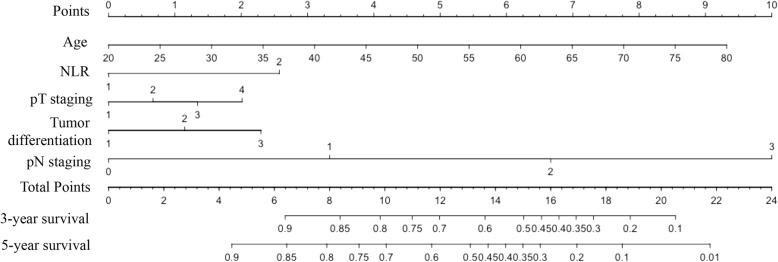
Postoperative prognostic nomogram predicted the probability of patients with resected NSCLC for 3- and 5-year overall survival. To use the nomogram, each patient was assigned a score on each variable axis, and the sum of these numbers could determine the location on total points axis. A line is drawn downward to the survival axes to determine the 3- or 5-year overall survival

A nomogram was established which embraced the significant prognostic factors, age, pathologic T and N staging, tumor differentiation, and neutrophil to lymphocyte ratio and had the ability to reflect the 5-year OS (in Fig. 3). The nomogram evinced that neutrophil to lymphocyte ratio made a significant contribution to survival outcomes.

### Internal validation and calibration plot

**Fig. 4:**
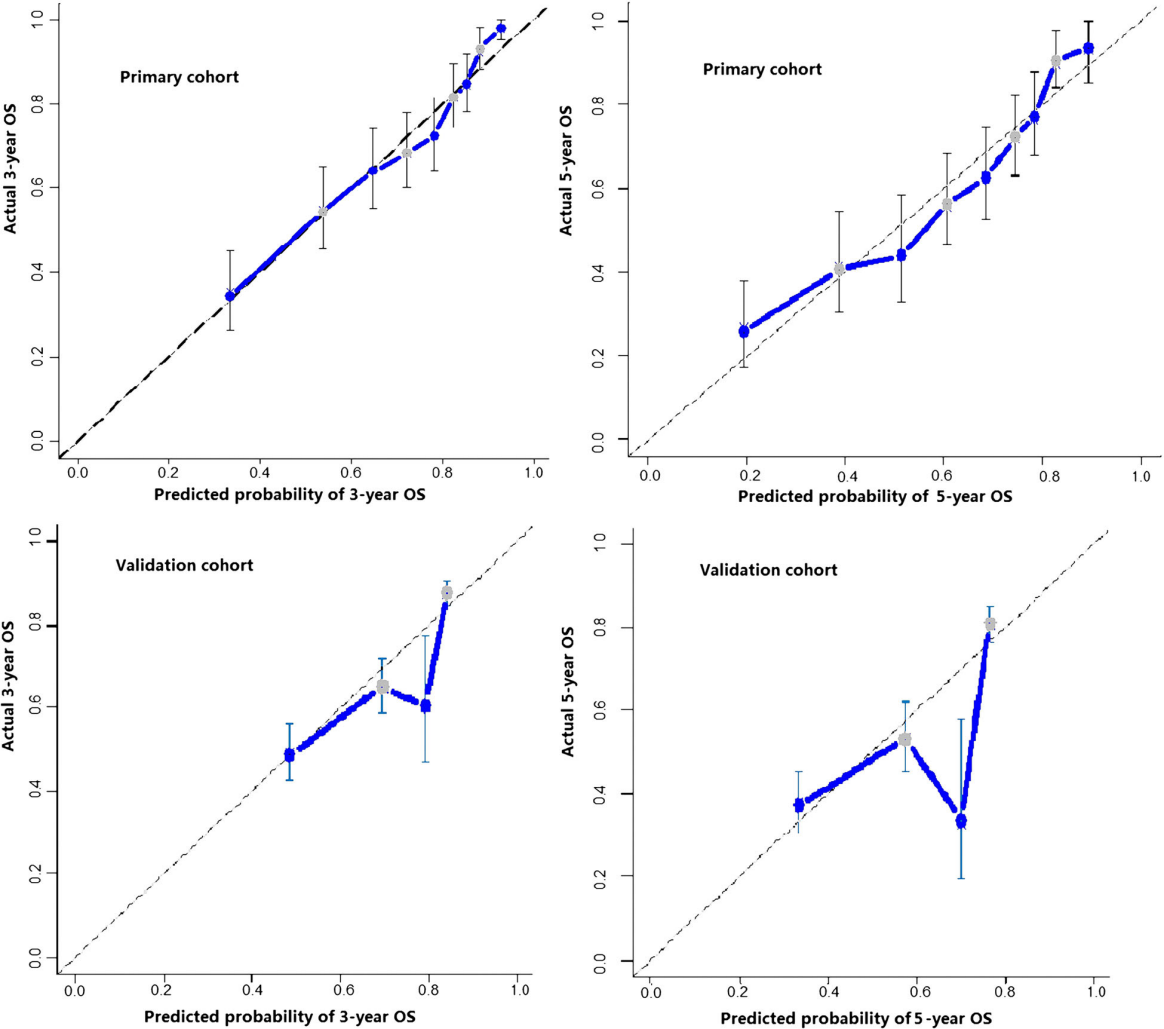
The calibration curves for predicting patient survival of 3- and 5-year OS in the primary cohort and Validation cohort

**Fig. 5:**
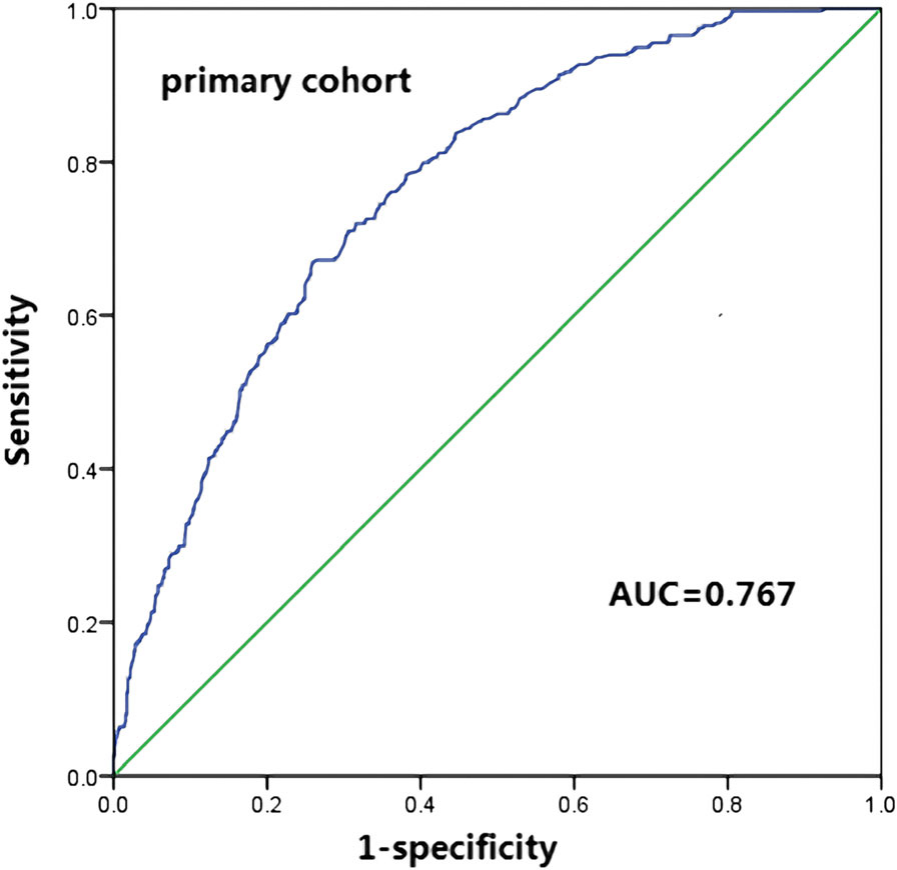
The AUC of the nomogram

The C-index was 0.72 in the nomogram, higher than that of TNM system staging (0.69). Afterwards, the 5-year OS calibration curves of our nomogram displayed an optimal agreement between the actual observed outcomes and the predictions (in Fig. 4), compared with TNM system staging. The nomogram of our model was validated by the sample size of 100, while TNM system staging was validated by the sample size of 300 for its fewer variates. In the same time, The ROC of the nomogram was performed and the AUC of our nomogram was 0.767 (Fig. 5).

## Discussion

Although there have been several nomograms used to select individual therapy for patients with lung cancer [16, 17], a nomogram incorporated with inflammatory response biomarkers has not been put forward. The aim of our study is to investigate the impact of inflammatory response biomarkers on survival outcomes and to establish an inflammation-related nomogram in patients with NSCLC who underwent surgery.

In our study, both classical and novel inflammatory response biomarkers are the candidates for nomogram including: neutrophil to lymphocyte ratio, absolute neutrophil counts to absolute lymphocyte counts, lymphocyte to monocyte ratio and platelet to lymphocyte ratio and systematic immune-inflammation index. All the biomarkers show their predictive ability on survival outcomes and among them, only the neutrophil to lymphocyte ratio has been selected to be included in the nomogram after survival analysis through Kaplan-Meier curves, univariate and multivariate method. For non-inflammatory biomarkers, pathologic T and N staging, age and tumor differentiation are also considered as independent prognostic factors which could be incorporated into the nomogram. In the nomogram, neutrophil to lymphocyte ratio is the third most important prognostic factors following pathologic N staging and age to predict the survival. Internal validation and calibration curve are performed to test the repeatability and reliability of the nomogram. Compared with TNM traditional system staging, the nomogram has a higher C-index (0.72) through internal validation, indicating that the nomogram has a better ability to discriminate survival outcomes. Calibration curves for the nomogram of 5-year OS disclose an excellent agreement between prediction and actual observation and is superior to those of TNM system staging. Based on the above results, we believe that inflammatory response biomarkers should be incorporated into the predictive models as independent prognostic factors of patients with lung cancer, and the inflammation-related nomogram have been shown to provide more precise prediction compared with traditional TNM classification.

Cancer-related inflammation has been referred as local inflammation and systemic inflammation which could promote tumorigenesis and metastasis [18] in a broad range of cancers [19]. Increasing novel inflammatory response biomarkers are therefore developed to better refine the stratification of patients. Recently, increasing attention is being paid to the biomarkers derived from innate immune cells in peripheral blood. Neutrophil to lymphocyte ratio is a simple index of the systemic inflammatory response, and the increased level of neutrophil to lymphocyte ratio has been shown to predict worse overall survival in patients with NSCLC [3, 4, 6–8]. Additionally, it has been reported that the perioperative use of nonsteroidal anti-inflammatory drugs (NSAIDs), such as celecoxib and ketorolac, could change the tumor microenvironment and reduce migration and invasion of circulating malignant cells [4, 20–22]. Taken together, these findings demonstrate the importance of perioperative inflammation and immune suppression on oncological outcomes.

Neutrophils could be stimulated to proliferate by cancer-related inflammatory factors, such as Tumor necrosis factor-alpha and Interleukin-6, which subsequently secrete reactive oxygen species and pro-angiogenic factors, and therefore favors tumorigenesis and tumor microenvironment [23, 24]. Also, bone marrow could lead to an abnormal release of neutrophils precursors upon inflammation. Regarding lymphocytes, they have shown to exert a vital role in cell-mediated immunity against host cancer cells, and the decreases in lymphocytes count have worse survival outcomes Nomograms possess their own merits of predicting oncologic prognosis, such as intuitive graphs and numerical probability of clinical events, so they are identified as reliable tools to quantify risks. Given the importance of neutrophils and lymphocytes in tumor development, we therefore seek to integrate the neutrophil to lymphocyte ratio into the nomogram for improving the accuracy of the predictive model. Our results indicate that the contribution of neutrophil to lymphocyte ratio is at the third place as a predictor, following pathologic N staging and age. Our proposed nomogram highlights the significant predictive role of neutrophil to lymphocyte ratio in prognosis.

Apart from inflammatory response biomarkers, age, tumor differentiation, pathologic T stage and pathologic N stage are the other independent prognostic factors which reveal a significant influence on survival. The nomogram incorporated with neutrophil to lymphocyte ratio might have the ability to predict the prognosis of patients undergoing surgery according to their inflammatory status, pathologic T and pathologic N stages and other tumor characteristics. Moreover, our nomogram could also assist clinicians in developing tailored treatment for individual patients based on their inflammatory status.

Our nomogram has some limitations. First, the analysis is conducted retrospectively which creates intrinsic drawbacks. Second, some prognostic parameters (such as carcinoembryonic levels) and other important molecular factors (such as Epithelial growth factor receptor mutation) are not included in our analysis due to lack of data.

## Conclusions

In conclusion, we have established an inflammation-related prognostic nomogram predicting individual survival in patients with NSCLC after surgery. Additionally, neutrophil to lymphocyte ratio can be considered as an independent prognostic factor. The proposed nomogram in this study provides better predictive accuracy and confirms the predictive value of inflammation response biomarkers. It offers a useful tool for providing reference for clinicians to assess the survival of individual patients after surgery.

## Abbreviations

ADC: Adenocarcinoma
dNLR: Derived neutrophil to lymphocyte ratio
LMR: Lymphocyte to monocyte ratio
NLR: Neutrophil to lymphocyte ratio
NSAID: Nonsteroidal anti-inflammatory drug
NSCLC: Non-small cell lung cancer
OS: Overall survival
PGTD: Pathological grading of tumor differentiation
PLR: Platelet to lymphocyte ratio
pN category: pathologcial N category
pT category: pathologcial T category
R: Reference
ROC: Receiver operating curve
SCC: Squamous cell carcinoma
SII: Systematic immune-inflammation index
